# In Vitro and In Silico Analyses of Nicotine Release from a Gelisphere-Loaded Compressed Polymeric Matrix for Potential Parkinson’s Disease Interventions

**DOI:** 10.3390/pharmaceutics10040233

**Published:** 2018-11-15

**Authors:** Pradeep Kumar, Yahya E. Choonara, Lisa C. du Toit, Neha Singh, Viness Pillay

**Affiliations:** Wits Advanced Drug Delivery Platform Research Unit, Department of Pharmacy and Pharmacology, School of Therapeutic Sciences, Faculty of Health Sciences, University of the Witwatersrand, Johannesburg, 7 York Road, Parktown 2193, South Africa; pradeep.kumar@wits.ac.za (P.K.); yahya.choonara@wits.ac.za (Y.E.C.); lisa.dutoit@wits.ac.za (L.C.d.T); neni2707@gmail.com (N.S.)

**Keywords:** alginate gelispheres, textural analysis, crosslinked matrices, PLGA discs, prolonged release, powder flow properties

## Abstract

This study aimed to develop a prolonged-release device for the potential site-specific delivery of a neuroprotective agent (nicotine). The device was formulated as a novel reinforced crosslinked composite polymeric system with the potential for intrastriatal implantation in Parkinson’s disease interventions. Polymers with biocompatible and bioerodible characteristics were selected to incorporate nicotine within electrolyte-crosslinked alginate-hydroxyethylcellulose gelispheres compressed within a release rate-modulating external polymeric matrix, comprising either hydroxypropylmethylcellulose (HPMC), polyethylene oxide (PEO), or poly(lactic-*co*-glycolic) acid (PLGA) to prolong nicotine release. The degradation and erosion studies showed that the produced device had desirable robustness with the essential attributes for entrapping drug molecules and retarding their release. Zero-order drug release was observed over 50 days from the device comprising PLGA as the external matrix. Furthermore, the alginate-nicotine interaction, the effects of crosslinking on the alginate-hydroxyethycellulose (HEC) blend, and the effects of blending PLGA, HPMC, and PEO on device performance were mechanistically elucidated using molecular modelling simulations of the 3D structure of the respective molecular complexes to predict the molecular interactions and possible geometrical orientation of the polymer morphologies affecting the geometrical preferences. The compressed polymeric matrices successfully retarded the release of nicotine over several days. PLGA matrices offered minimal rates of matrix degradation and successfully retarded nicotine release, leading to the achieved zero-order release for 50 days following exposure to simulated cerebrospinal fluid (CSF).

## 1. Introduction

Parkinson’s disease (PD), a central nervous system disorder, is associated with difficulties with respect to movement, typically outlined by the patient’s shuffling gait due to unstable posture, bradykinesia, tremor, and rigidity. The progressive neuronal loss in PD is prolonged, requiring clinical neuroprotection and/or disease modification as therapeutic strategies for effectively reducing PD-related disability. The potential application of nicotine as a neuroprotectant in PD was demonstrated as far back as 1926 [1]. Nicotine has previously been shown to protect against the degeneration of nigrostriatal neurons via evoking the release of dopamine from the striatum [2,3,4,5]. PD is still an incurable disease. The available therapeutic options only offer symptomatic relief and primarily aim to improve the functionality of the patient with no intervention towards the progression of the associated neurodegeneration. Additionally, a major challenge is to improve patient compliance to therapy by reducing the side-effects associated with continuous multiple dosing via the oral route subsequently leading to erratic plasma drug levels.

Generally, prolonged parenteral zero-order drug delivery systems have the capacity to minimize dose-related side-effects owing to a constant and sustained drug release profile. This is applicable specifically to drugs with narrow therapeutic indices, wherein such systems can provide a reduction in administered dose, avoidance of fluctuations in plasma drug levels, reduced frequency of administration, and hence the enhancement of patient compliance [6,7]. The use of polymers is a popular means of achieving controlled drug release due to the simplicity, cost-effectiveness, ease of manufacturing, versatility, and the ability to deliver compounds with a wide range of solubilities [8]. 

Hydroxypropylmethylcellulose (HPMC) and polyethylene oxide (PEO) have been used extensively to formulate controlled-release drug delivery systems. Both polymers are hydrophilic and undergo simultaneous swelling and erosion when exposed to hydration. Depending on the solubility of incorporated drugs within monolithic matrices of such polymers, the actives diffuse out into the bulk medium preceding or following erosion of the polymer [9,10]. The mechanisms relating to the swelling and hydration of hydrophilic polymeric matrices may include, but are not limited to, polymeric chain extension, disentanglement, and solvent accommodation to macroscopic characteristics such as drug release [11,12]. In addition, formulation factors such as the quantity of drug loading, drug-polymer ratio, drug particle size and molecular mass, polymer viscosity and molecular weight, and presence of formulation excipients and release modulators significantly affect the drug release rate from hydro-swelling matrices [13,14]. Poly(lactic-*co*-glycolic acid) (PLGA), a polyhydroxy acid polymeric carrier, is capable of delivering drugs as a controlled-release site-specific system and has the advantage of being moderately aqueous-soluble for controlled drug diffusion [15]. Biocompatibility studies have indicated that PLGA is biodegradable and considerably well tolerated when implanted into the CNS [16]. Consequently, no surgical intervention or procedures are required for removal of implantable devices prepared with PLGA. Fournier and co-workers (2003) established that regardless of the implantation site, PLGA devices initiate only a moderate and non-specific inflammatory reaction, mainly due to mechanical insult during the implantation procedure [16].

The purpose of this study was to incorporate nicotine-loaded alginate-hydroxyethycellulose (HEC) gelispheres into an external polymeric matrix with the aim of modulating drug release to achieve prolonged zero-order release. The alginate-HEC gelispheres were previously developed by the authors and were physically crosslinked using divalent ions [17]. HPMC, PEO, and PLGA were selected as the polymers for formulating the device due to the desirable inherent characteristics of the polymers for implantation into the CNS. The study was further aimed to elucidate the molecular mechanisms inherent to the drug-polymer interactions, formation of the gelispheres, and finally the fabrication and performance of various composite polymeric matrices using static lattice atomistic simulations. 

## 2. Materials and Methods 

### 2.1. Materials

Nicotine was obtained from Sigma-Aldrich, St. Louis, MO, USA. The polymers employed for gelisphere preparation included sodium alginate (FMC BioPolymer, Drammen, Norway) and HEC (Hercules Inc., Natrosol^®^ Pharm, Wilmington, DE, USA). The crosslinking reagents employed were calcium chloride (CaCl2) and barium chloride (BaCl2) (UniLab^®^, Saarchem (Pty) Ltd., Krugersdorp, South Africa). PLGA (Resomer^®^ L503, Evonik Nutrition & Care GmbH, Essen, Germany), HPMC (Methocel™ K4M, Dow Chemical Company, Pittsburg, CA, USA) and PEO (PolyoxTM WSR 303; Union Carbide, Bound Brook, NJ, USA) were employed as the external matrices.

### 2.2. Methods

#### 2.2.1. Formulation of the Reinforced Alginate-HEC Gelispheres

The alginate-HEC gelispheres were formulated employing ionotropic gelation as described elsewhere by the authors [17]. Briefly, an alginate (2% *w*/*v*) and HEC (1% *w*/*v*; the reinforcing agent) dispersion was prepared in double-deionised water and subsequently homogenized. Thereafter, nicotine (1% *w*/*v*) was added to the polymeric dispersion through titration at a rate of 5 mL/min. The resulting solution was immediately added into the crosslinking solution comprising BaCl_2_ and CaCl_2_ (2% *w*/*v*). The crosslinked alginate-HEC (ALG-HEC) gelispheres were crosslinked for 30 min and thereafter washed with deionized water. Formulations were then exposed to 2 M HCl subsequent to curing in order to precipitate alginic acid in the crosslinked system and retard the hydration and swelling dynamics of the gelispheres.

#### 2.2.2. Formulation of the Gelisphere-Loaded External Polymeric Matrices 

Briefly, 500 mg of a dry polymer mixture comprising either HPMC, PEO, PLGA, or various binary combinations of the polymers and gelispheres equivalent to 20 mg of entrapped nicotine (approximately 100 mg gelispheres) were compressed into 10-mm flat-faced discs (Beckman^®^ Hydraulic Press; Beckman Instruments Inc., Fullerton, CA, USA), applying a compression force of 8 tons. Table 1 summarizes the compositions of the various formulations investigated. 

#### 2.2.3. Evaluation of the Flowability and Friability of the Polymeric Material 

To evaluate the flowability of the powders the angle of repose (*φ*) method was employed. A conical funnel was attached to a retort stand with the bottom orifice (diameter 7 mm) at a 10-cm height above the horizontal surface. Samples of 3 g of powder were placed into the funnel while keeping the orifice closed. The center-point and a graduated set of axes was marked on a piece of graphical paper and placed beneath the funnel. The contents of the funnel were allowed to flow through the orifice and the angle of repose (*φ*) was calculated employing Equation (1):

tan*φ* = 2*h*/*D* (1)

where *h* is the powder height at center-point (mm) and *D* is the diameter of powder bed formed (mm).

Friability analysis evaluated the ability of the compressed gelisphere-loaded matrices to withstand mechanical damage due to the variation in particle size. The matrix friability was measured using a Roche^®^ Friabilator (Hoffman la Roche, Basel, Switzerland). Samples were accurately weighed and placed into the friabilator (*N* = 3). Rotation times of 4 min and 20 min at 25 rpm were used. Matrices were then removed and the surface was lightly dabbed to remove any loose particles and re-weighed. The mass loss was calculated employing Equation (2).

*WL*_t_ = (*W*_0_ − *W*_t_)/*W*_0_ × 100 (2)

where *WL*_t_ is the mass loss at time *t* (%), *W*_t_ is the mean mass at *t*; and *W*_0_ is the mean mass at time *t*_0_.

#### 2.2.4. Determination of the Brinell Hardness Number of the Compressed Polymeric Matrices

The change in physicomechanical properties of the matrices following exposure to simulated cerebrospinal fluid (CSF) was assessed by measuring the Brinell Hardness Number (BHN). The BHN is an indication of the force required to indent the surface of the gelisphere-loaded matrices and represents the surface hardness of the compressed polymeric matrices. A TA.XTplus Texture Analyser (StableMicrosystems^®^, Surrey, UK) was used to generate force-distance profiles for matrices exposed to simulated CSF over predetermined time intervals (pH 7.4; 37 °C). The input parameters employed with a 5-kg load cell were pre-test speed (1.0 mm/s), test speed (0.5 mm/s), post-test speed (1.0 mm/s), and trigger force (0.5 N). The BHN (N/mm^2^) was calculated employing Equation (3).

(3) $$BHN=\frac{2F}{\pi D\;\left[D-{\left(D^{2}-d^{2}\right)}^{\frac{1}{2}}\right]}$$

where *D* is the diameter of the ball probe (3.175 mm), *d* is the indentation distance (0.25 mm), and *F* is the force (N) generated. 

#### 2.2.5. Evaluation of Conductivity Changes Following Polymeric Matrix Gelation

An OAKTON TDS Test^TM^ Kit (Model WD-35661-70; OAKTON Instruments, Solon, OH, USA) was employed to measure the conductivity of the PBS (0.1 M; pH 7.4) solution containing the polymeric discs at predetermined intervals of time over a period of 30 days. Samples were extracted from the solutions and diluted 1:10 in double deionized water. The electrode was immersed in the diluted polymer-PBS solution and conductivity (µSiemens) was read.

#### 2.2.6. Evaluation of the Matrix Erosion upon Hydration

Pre-weighed hydrated discs were removed from simulated CSF at predetermined intervals and dried under ambient conditions (21 °C) for 7 days. The dried discs were re-weighed and the percentage weight loss (% WL) calculated. 

#### 2.2.7. Evaluation of Surface Morphology of the Matrices

Scanning electron microscopy (SEM) was employed to assess the changes in the porosity of the polymer matrices following hydration. Dried samples of the discs were cross-sectioned and coated with a thin layer of colloidal graphite. Thereafter they were mounted onto aluminum stubs and coated with a thin layer of gold–palladium under an electrical potential of 20 keV. Samples were viewed in the FE-SEM (JEOL JSM-840, Tokyo, Japan) at a magnification of 65 and probe current of 30 nanoamps. SEM images were processed using Mathematica^TM^ 8.0 (Wolfram Research, Champaign, IL, USA) as described in the supplementary data. Initially, images were cropped to restrict image content to that of the scaffold.

#### 2.2.8. Textural Analysis of the Hydrated and Unhydrated Gelisphere-Loaded External Polymeric Matrices 

The Texture Analyzer (TAXT.plus, Stable Micro Systems^®^, Surrey, UK) was employed to conduct textural analysis of the gelisphere-loaded polymeric matrices (*N* = 5). The texture analyzer was fitted with a 10-mm cylindrical steel probe and a 40-kg load cell was used. Matrix resilience, deformability modulus, and fracture energy of the crosslinked gelispheres were assessed according to their G and M content in both the unhydrated and hydrated states. For the later test, gelispheres were immersed in 15 mL double deionised water in glass vials and agitated at 50 rpm in a shaker bath (Labline^®^ Instruments, Melrose Park, IL, USA) maintained at 37 °C. Samples (*N* = 10) were removed and analyzed for four 24-h intervals and evaluated for the above stated entities. The association of the monomers, i.e., GG, GM, and MM blocks and their interaction with the crosslinking cations on the mechanical properties was also examined. Samples with an identical G/M ratio were compared according to differences in viscosity. The input parameters employed with a 5-kg load cell (50% strain) were pre-test speed (1.0 mm/s), test speed (0.5 mm/s), post-test speed (1.0 mm/s), and trigger force (0.5 N). The force-time profile was employed for the determination of matrix resilience. This refers to the ability of the gelisphere to recover upon application of deforming stress to its original state of equilibrium. This was calculated as the ratio of the areas under the compression and decompression curve. A forcedistance profile generated for the gelispheres was employed to obtain the area under the curve (AUC), which enabled the evaluation of the energy needed to rupture the gelisphere matrix, i.e., the fracture energy (Nm) while the gradient to the peak force, known as the deformability modulus (N/mm), corresponds to the deformability modulus of the gelisphere matrix [18]. 

#### 2.2.9. In Vitro Drug Release Studies of the Gelisphere-Loaded External Polymeric Matrices 

In vitro drug release studies were performed on the nicotine-loaded matrices using simulated cerebrospinal fluid (CSF). Gelisphere-loaded external polymeric matrices were immersed in 100 mL of 0.1 M phosphate-buffered saline (PBS) (pH 7.4; 37 °C) in sealed 150-mL glass jars that were placed in a shaker bath (Labex, Stuart SBS40 South Africa) and agitated at 50 rpm. At predetermined intervals, 5-mL samples were removed and the samples were analyzed by UV spectroscopy (245 nm; Specord^®^ 40, Analytik Jena UV-VIS, Aargau, Switzerland). To maintain sink conditions; 5 mL of nicotine-free PBS were replaced into the glass jars.

#### 2.2.10. Static Lattice Atomistic Molecular Structural Simulations

To elucidate the interaction profile of individual molecules within the alginate-drug complex, alginate-HEC gelisphere matrices, and the external matrices, molecular mechanics computations were conducted in a vacuum using the HyperChem^TM^ 8.0.8 Molecular Modeling System (Hypercube Inc., Gainesville, FL, USA) and ChemBio3D Ultra 11.0 (CambridgeSoft Corporation, Cambridge, UK). The polymers (PLGA and PEO) were drawn in their syndiotactic stereochemistry as 3D models; the carbohydrate (alginate) and cellulose derivatives (HEC and HPMC) were built from standard bond lengths and angles using a sugar builder module; and the structure of nicotine was drawn with natural bond angles. The structures so-obtained were energetically optimized using a progressive minimization paradigm employing MM+ and the Amber 3 (Assisted Model Building and Energy Refinements) force field. The conformer having the lowest energy was used to create the polymer-polymer complexes. A complex of one molecule with another was assembled by disposing them in a parallel way, and the same procedure of energy minimization was repeated to generate the final models: alginate-nicotine (ALG–NCT), ALG–HEC, ALG–HEC–Ca^2+^, ALG–HEC–Ba^2+^, PLGA–HPMC, HPMC–PEO, PLGA–PEO, and PLGA–HPMC–PEO. Full geometry optimization was carried out in vacuum employing the Polak–Ribiere conjugate gradient algorithm until an RMS gradient of 0.001 kcal/mol was reached. For molecular mechanics calculations in vacuum, the force fields were utilized with a distance-dependent dielectric constant scaled by a factor of 1. The 1–4 scale factors were electrostatic = 0.5 and van der Waals = 0.5 [19]. 

## 3. Results and Discussion

### 3.1. Flowability and Friability of the Polymeric Matrices

The flowability results indicated that PLGA had the greatest degree of inter-particle interaction (cohesion). Hence, its high angle of repose (AR) (40.40°) indicated that it had poor flow properties. When combined with HPMC, their mutually attractive interaction was indicated by the highest AR of 52.41°, which implies that the combination had the potential for producing highly dense compressed discs. PEO on the other hand indicated excellent flow properties, as it demonstrated a lower AR of 13.49°. This can be explained in terms of the fact that it was predominately composed of spherical granular particles. These particles had a smaller surface area per particle to interact with each other, as compared to PLGA and HPMC. Its incorporation with PLGA and HPMC resulted in a significant decline in AR (11.46° and 12.74°, respectively) and improved overall flowability, and thus it negatively impacted on the cohesive tendency of the particles, which implied that matrices generated with the aforementioned combinations would generate less compact matrices. From a manufacturing process point of view, improved flowability permits easier processing conditions. However, this implies that additional substances (such as binders) may need to be added to enhance powder compressibility to generate discs. According to the United States Pharmacopoeia (USP 23), conventional compressed tablets that incur a loss in weight after 100 revolutions (which is the equivalent of 4 min in the friabilator at 25 rpm) in a friabilator of less than 0.5–1.0% are considered acceptable. The results indicated that the discs generated were well within the predetermined limits. PLGA discs were exceedingly well compressed and underwent negligible loss in weight following 500 revolutions (i.e., 20 min at 25 rpm) (Table 1). Furthermore, the incorporation of PLGA with HPMC and PEO into the disc resulted in an enhanced robustness imparted into the matrix, while the addition of PEO has the reverse effect, indicated by the greater friability. 

### 3.2. Changes in the Brinell Hardness Number (BHN) of Compressed External Polymeric Matrices Following Hydration

**Figure 1:**
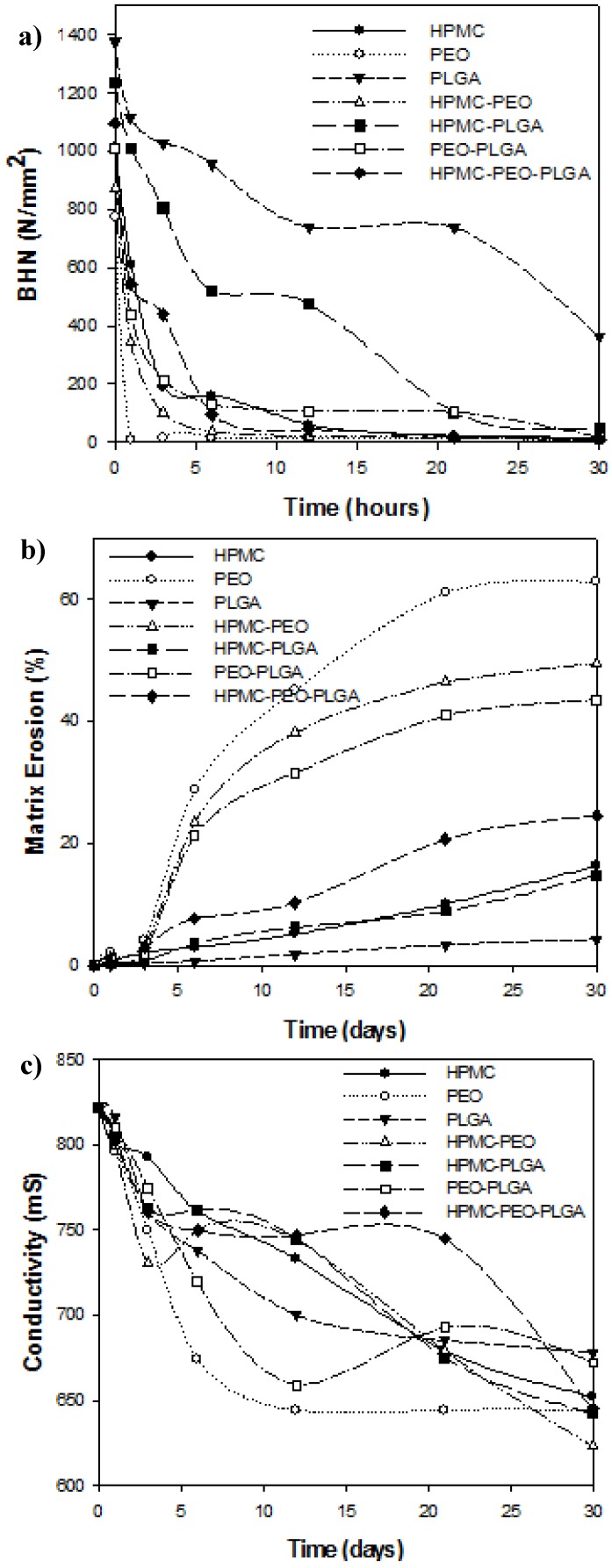
Physical properties of compressed polymeric discs following exposure to simulated CSF over a period of 30 days: (**a**) Changes in the Brinell Hardness Number (N/mm^2^; *N* = 3; SD < 23.67 N/mm^2^); (**b**) Matrix erosion (%; *N* = 3; SD < 0.05%); and (**c**) Changes in conductivity (µSiemens; *N* = 3; SD < 16.34 µS).

Evaluating changes in the Brinell Hardness Number (BHN) of the discs allowed us to establish the rate at which water penetrated the surface of the discs and lead to their subsequent hydration and erosion. The faster this occurred, the more quickly the incorporated gelispheres became exposed to the surrounding aqueous medium and released the entrapped nicotine. The results demonstrated that the most significant changes in BHN occurred within the first day of exposure to simulated CSF (Figure 1a). Discs composed of PEO in particular experienced a significant decline in BHN value within the first 24 h (Figure 1a). Both PEO and HPMC hydrogels imbibed and retained a substantial amount of water from the surrounding buffer media to become hydrated, and subsequently underwent polymeric relaxation, generating a gel front on the surface of the disc. Subsequent to the initial 24-h decline, the discs underwent gelation to the core of their compressed matrices rapidly. Furthermore, the presence of PEO impeded cohesion between the HPMC and PLGA powder particles consequently generated matrices that were more prone to degradation upon exposure to hydration and mechanical stress. Due to the more complex nature of the HPMC polymer in comparison to PEO, it did not undergo polymeric relaxation as rapidly. Thus, the BHN value for HPMC declined most significantly between days 1 and 6. PLGA proved to be the exception to this rule. It demonstrated a steady decline in BHN over the 30 days. The combination of PLGA–HPMC also depicted a gradual, albeit more rapid decline in BHN, indicating that the presence of PLGA with the HPMC generated sufficient cohesion between the polymers to restrict the diffusion of water molecules into the compressed polymeric matrix.

### 3.3. Evaluation of Erosion of Compressed External Polymeric Matrix

In contact with dissolution medium (in this case simulated CSF), hydrophilic polymers such as PEO and HPMC either swell into a hydro-gel layer or undergo erosion or both. Such swelling behavior is proportional to the rate of hydration and along with relative mobilities of dissolution medium and drug it dictates the kinetics as well as mechanism of drug release [20]. As portrayed in the previous results, PEO discs demonstrated lower gel strength, greater water uptake, and therefore demonstrated greater matrix erosion as depicted in Figure 1b. HPMC discs on the other hand demonstrated greater matrix swelling with negligible matrix erosion (Figure 1b). However, from the results it was observed that the rate of drug release by far superseded the rate of matrix degradation; hence it was concluded that the rate of drug release in this case was predominately a result of the diffusion of the drug from the matrices as opposed to matrix degradation. 

As mentioned previously, PLGA is a hydrophobic bioerodible polymer. Thus, the mechanics of polymeric degradation and matrix erosion therefore occur via two main mechanisms, namely bulk erosion and surface erosion [21]. The degradation of such polymers is based on the conversion of the macromolecule into its water-soluble monomers via the hydrolytic cleavage of the ester bonds that constitute the backbone of the polymer. While bulk erosion does occur, a second mechanism which is surface erosion also takes place concurrently. This refers to the occurrence of hydrolytic breakdown of the polymer confined to the surface of the polymeric matrix. This was reflected by the steady decline in its BHN value over time following hydration (Figure 1b). Drug release therefore involved complex combination of both the drug diffusion from the surface as a result of bulk erosion as explained further in the manuscript.

### 3.4. Changes in Conductivity of Compressed External Polymeric Matrices Following Hydration

These results inversely correlated with those observed for matrix erosion i.e., the greater the matrix erosion, the lower the observed conductivity of the solution. This indicated a greater degree of interaction between the untangling polymeric chains and the ions in solution. As greater quantities of the polymer unraveled from the disc, i.e., the matrix eroded, it ionized and interacted with ions in the surrounding PBS. Hence, we observed a decline in the conductivity of the solution over the 30 days (Figure 1c). The greatest decline was observed for PEO. This is not surprising as it undergoes the most rapid rates of matrix degradation. Since the most significant phase of degradation of the PEO discs occurred within the first 12 days of exposure to simulated CSF, conductivity declined exponentially until this point, after which equilibrium was attained. HPMC demonstrated a steady, almost linear decline in conductivity until day 21, after which a significant amount of the matrix had degraded to interact with surrounding counter-ionic species. As expected, PLGA expressed the least change in electrical conductivity, since it demonstrated the minimal change in weight (matrix erosion) over time following hydration.

### 3.5. Evaluation of Porosity Changes upon Hydration

**Figure 2:**
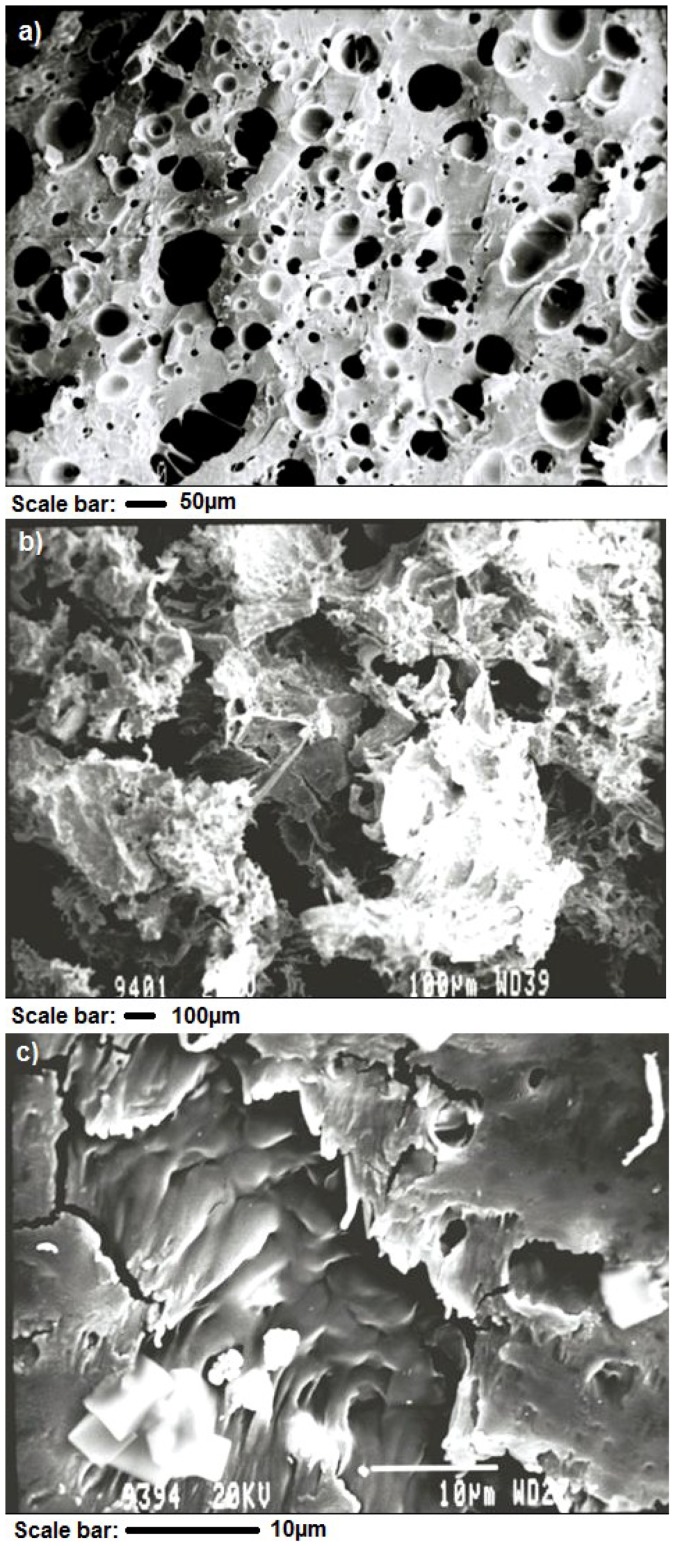
Scanning electron micrographs (Magnification 65×) of compressed: (**a**) PLGA, (**b**) HPMC–PLGA, and (**c**) PEO-PLGA discs following exposure to simulated cerebrospinal fluid (CSF) after 21 days.

Scanning electron micrographs of the discs demonstrated the progression of pore formation and enlargement, formation of a trabeculated network of channels within the structure followed by erosion, and loss of integrity (Figure 2). PLGA discs demonstrated the most intact matrices in comparison to other systems. Figure 2a indicates the formation of a porous structure within the matrix at day 21 following exposure to simulated CSF. Larger pores (diameter of approximately 300 µm) were limited to the periphery of the discs, while the core of the matrix was relatively intact (where pores have a diameter of approximately 50 µm). 

This matrix did not expand significantly, nor did it undergo any significant erosion. In contrast by day 21, HPMC–PLGA discs (Figure 2b) had a significantly more patent network, with pore sizes in the core of the matrix having comparatively larger diameters. The disc expanded in width and cross-sectional diameter due to the tendency of HPMC to ‘swell’. PEO–PLGA discs (Figure 2c) demonstrated a significant degree of erosion within the matrix while simultaneously indicating a greater degree of swelling and erosion. By day 21, the entire network, including the core of the matrix, was extremely patent and highly trabeculated. The majority of the matrix appeared to be composed of PLGA. A detailed image processing analysis of the PLGA matrix is provided in the supplementary data (Figure S1).

### 3.6. In Vitro Drug Release from Nicotine-Loaded Compressed Gelisphere Polymeric Matrices 

**Figure 3:**
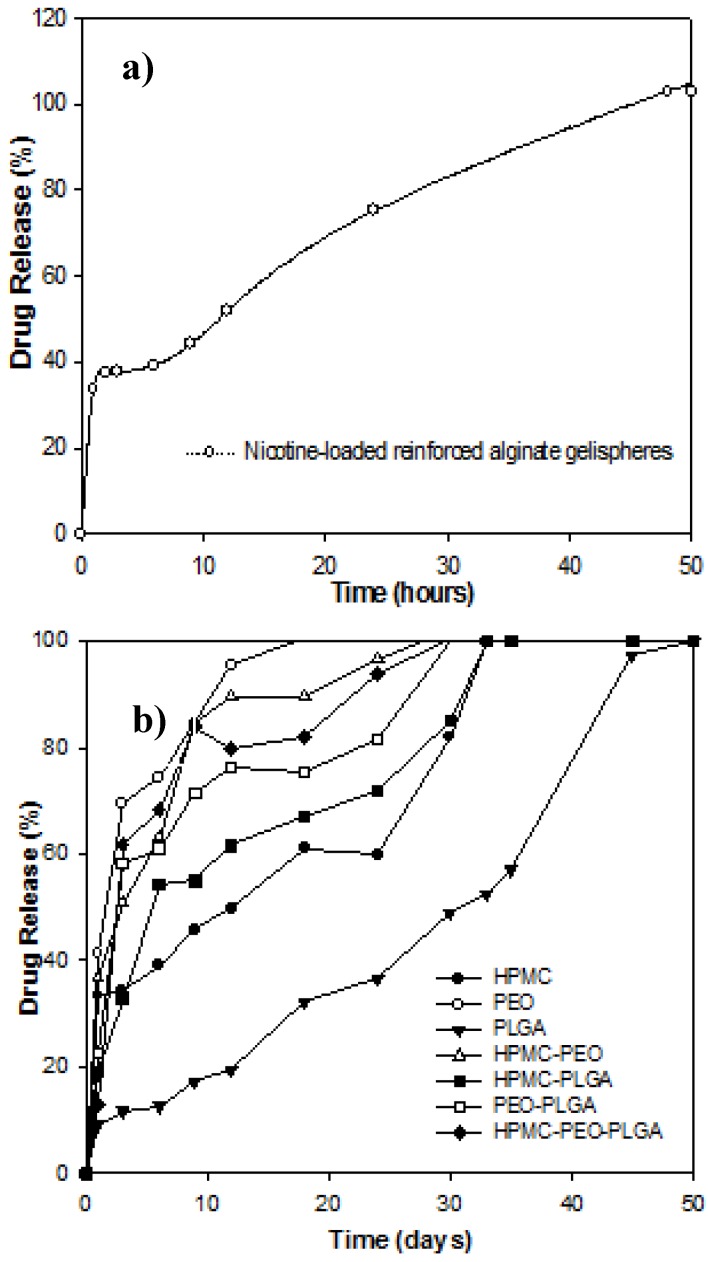
Drug released (%) from: (**a**) nicotine-loaded reinforced alginate gelispheres, and (**b**) nicotine-loaded reinforced alginate gelispheres compressed into polymeric discs following exposure to simulated cerebrospinal fluid (CSF) over a period of 50 hours (*N* = 3; SD < 0.53) and 50 days (*N* = 3; SD < 0.59%) respectively.

In vitro drug release studies in simulated CSF indicated that reinforced alginate gelispheres released 100% of entrapped drug within 50 h through a biphasic first- and zero-order release profile (Figure 3a). It was observed that reinforced alginate gelispheres incorporated into PLGA discs demonstrated zero-order release kinetics that were able to provide controlled and prolonged release for approximately 50 days (Figure 3b). Drug release kinetics indicated a rapid burst in drug release after day 35, where after a first-order release profile was obtained. An additional 40% of entrapped nicotine was released between days 35 and 40. In retrospect it was observed that PLGA had the maximum cohesive tendency between its particles and has the ability to be most compressible. Being a hydrophobic polymer, its main degradative mechanisms are through the hydrolytic cleavage of its polymeric backbone. Consequently, these discs underwent minimal matrix degradation. 

The rapid release rates observed from PEO and HPMC matrices (Figure 3b) allowed us to conclude that drug release occurred primarily by Fickian diffusion. The presence of PEO in matrices enhanced the rate of drug release from the compressed discs. Its hydrophilic character allowed rapid disentanglement and degradation of the compressed matrices. Its tendency was therefore to undergo comparatively rapid bulk erosion when it came in contact with the alkaline hydrating medium (Figure 3b). HPMC is a hydrogel; hence, it has the capacity to imbibe a significant amount of water before beginning to degrade. Furthermore, as observed from previous results, its particles were sufficiently cohesive to generate compact discs that are able to provide zero-order release following the achievement of an equilibrium hydrated state (discussed under later sections). Between days 3 and 18, a linear drug release profile was observed from HPMC discs (Figure 3b). Prior to this, a burst release was observed, whereby approximately 33.25% of the entrapped nicotine was released within the first 24 h. Combining HPMC and PLGA had a unique effect on drug release. While in this case zero-order release was also observed, they exceeded those observed with the individual polymers. A burst effect was observed until day 6, by which time 54.31% of the drug had been released (Figure 3b). Thereafter, drug release followed a linear zero-order release profile until day 32. Release kinetics for all other combinations exhibited rapid burst rates of drug release and predominately first-order release (Figure 3b). 

### 3.7. Molecular Mechanics Energy Relationship (MMER) Analysis

Molecular mechanics energy relationship (MMER), analysis, a method for analytico-mathematical representation of potential energy surfaces, was used to provide information about the contributions of valence terms, noncovalent Coulombic terms, and noncovalent van der Waals interactions for the drug-polysaccharide complex, crosslinked polysaccharide morphologies, and the polymer-polymer composites. The MMER model for the potential/steric energy factors in various molecular complexes can be written as:

*E*_molecule/complex_ = *V*_∑_ = *V*_b_ + *V*_θ_ + *V*_φ_ + *V*_ij_ + *V*_hb_ + *V*_el_ (4)

where *V*_∑_ is related to total steric energy for an optimized structure, *V*_b_ corresponds to bond stretching contributions, *V*_θ_ denotes bond angle contributions, *V*_φ_ represents the torsional contribution from dihedral angles, *V*_ij_ incorporates van der Waals interactions due to non-bonded interatomic distances, *V*_hb_ symbolizes hydrogen bond energy function, and *V*_el_ stands for electrostatic energy.

#### 3.7.1. Energy Minimizations Involving Drug-Polymer Morphologies

**Figure 4:**
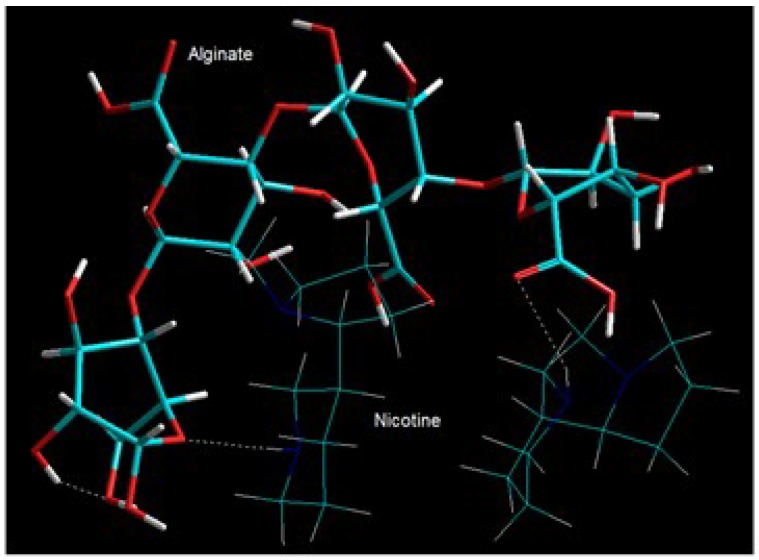
Visualization of geometrical preferences of nicotine in complexation with alginate after molecular mechanics simulations. Color codes: C (cyan), O (red), N (blue), and H (white).

The energy-minimized and geometrically optimized conformation depicting the alginate–nicotine (ALG–NCT) complex is presented in Figure 4. The component binding energies and intrinsic molecular attributes are listed in Equations (5)–(7) and Table 2, respectively. It is evident from Figure 4 that NCT and ALG show close interaction via formation of H-bonds between C–O–C...N–H and C=O...N–H group of ALG and NCT, respectively. The final energy of the drug–polymer complex was stabilized by all three non-bonding interactions (van der Waals interactions, H-bonding and electrostatic interactions) between the constituent molecules ALG and NCT. The surface-to-volume ratio (SVR) of the complex ALG–NCT (SVR = 0.451) displayed lower SVR than the individual molecules (Table 2) further confirming the stability of the complex—the lower the SVR, the more stable the structure. However, the finally globally minimized energy values suggest that the ALG–NCT complex is destabilized by a binding energy of ~4 kcal/mol (Equations (5)–(7)) due to exceptionally high torsional contribution arising due to deviations from optimum dihedral angles (Equation (7)). Such interactions may induce dipoles in the molecular complex due to the torsional strains experienced around dihedral angles.

*E*_ALG_ = −23.369*V*_∑_ = 2.148*V*_b_ + 26.810*V*_θ_ + 23.631*V*_φ_ + 8.649*V*_ij_ − 4.500*V*_hb_ − 80.110*V*_el_ (5)

*E*_NCT_ = 13.283*V*_∑_ = 0.388*V*_b_ + 3.672*V*_θ_ + 6.198*V*_φ_ + 3.023*V*_ij_ (6)

*E*_ALG-NCT2_ = 7.204*V*_∑_ = 3.403*V*_b_ + 34.575*V*_θ_ + 54.275*V*_φ_ + 8.182*V*_ij_ − 5.407*V*_hb_ − 87.824*V*_el_ (7)

[Δ*E*_BINDING_ = 4.007 kcal/mol]

It can be deduced from the above discussion that although nicotine formed a polyelectrolyte complex in association with alginate, the complex being unstable as it was might have led to release of nicotine upon hydration of the matrix. The above findings corroborated with the need of strategies for controlling the release of nicotine from the alginate matrix. We propose that this may be achieved by three different approaches applied together. Firstly, this may be achieved by inclusion of another polymer to stabilize the alginate matrix. Secondly, the strengthening of the alginate matrix can be achieved by crosslinking the hydrogel matrix using divalent ions such as Ca^2+^ or Ba^2+^. The third approach is by incorporating the crosslinked blend hydrogel–matrix in a release rate-modulating external polymeric matrix.

#### 3.7.2. Mechanistic Elucidation of Crosslinked Polymer Conformations

**Figure 5:**
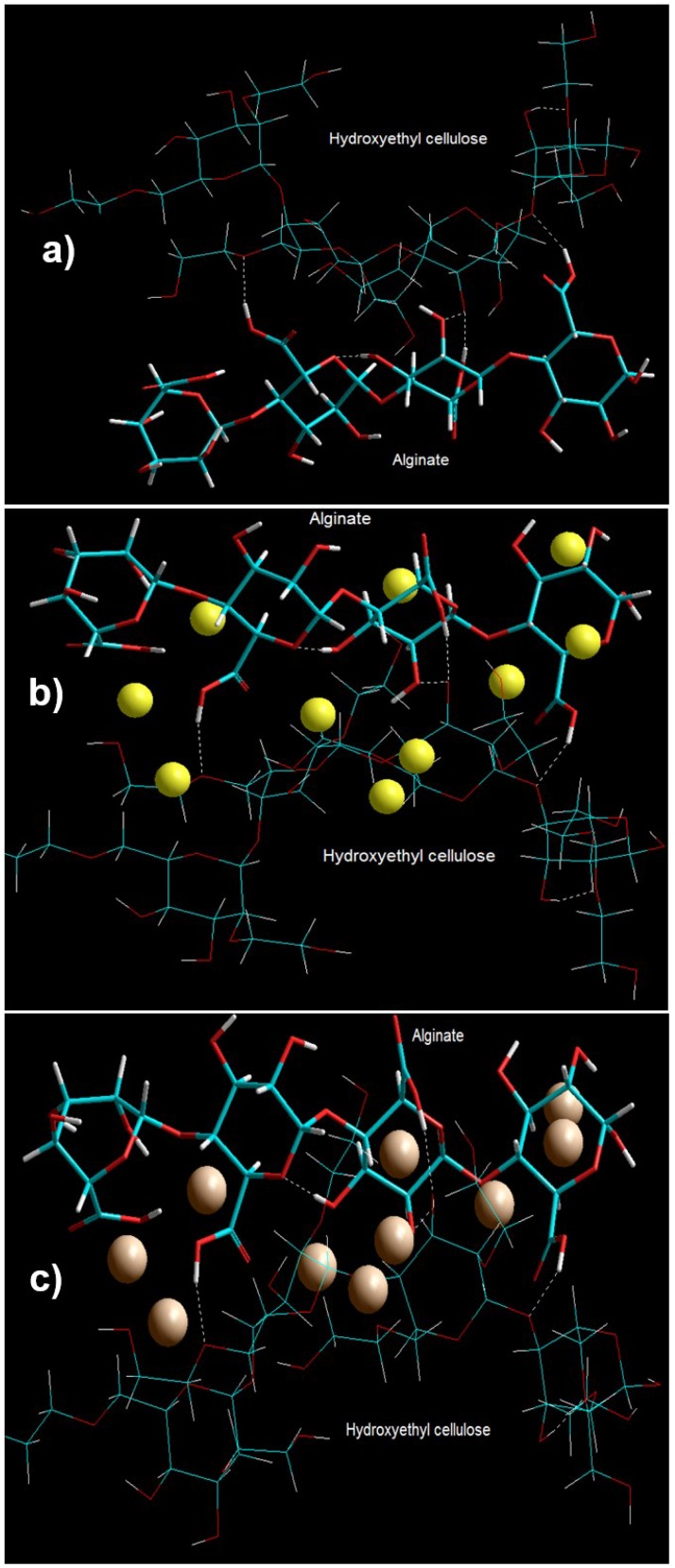
Visualization of geometrical preference of (**a**) Hydroxyethyl cellulose in complexation with alginate; (**b**) oligosaccharide–Ca^2+^ complex, and (**c**) oligosaccharide–Ba^2+^ complex derived after molecular mechanics simulations. Color codes: C (cyan), O (red), N (blue), Ca (yellow), Ba (brown), and H (white).

The general molecular mechanics program was used in this study to compute the energy of the polymer–saccharide (ALG–HEC) and the cation-polymer-saccharide conformations. The geometrical conformation of the ALG–HEC after SLAS are shown in Figure 5a and the corresponding energy attributes are depicted in Equations (5), (8) and (9).

*E*_HEC_ = 130.862*V*_∑_ = 4.017*V*_b_ + 80.816*V*_θ_ + 35.115*V*_φ_ + 10.28*V*_ij_ − 0.231*V*_hb_ + 0.863*V*_el_ (8)

*E*_ALG–HEC_ = −4.459*V*_∑_ = 6.685*V*_b_ + 44.109*V*_θ_ + 77.607*V*_φ_ + 9.085*V*_ij_ − 4.062*V*_hb_ − 137.886*V*_el_ (9)

[Δ*E*_BINDING_ = −111.952 kcal/mol]

*E*_ALG–HEC–Ca2+_ = −10.907*V*_∑_ = 6.666*V*_b_ + 44.137*V*_θ_ + 77.733*V*_φ_ + 2.794*V*_ij_ − 4.018*V*_hb_ − 138.22*V*_el_ (10)

[Δ*E*_BINDING_ = –118.4 kcal/mol]

*E*_ALG–HEC–Ba2+_ = −13.641*V*_∑_ = 6.700*V*_b_ + 44.245*V*_θ_ + 77.981*V*_φ_ + 0.116*V*_ij_ − 4.060*V*_hb_ − 138.625*V*_el_ (11)

[Δ*E*_BINDING_ = −121.134 kcal/mol]

The final minimized energy values for the ALG-HEC complex demonstrated a stabilization of binding energy by 111.952 kcal/mol and was significantly supported by the van der Waals interactions due to the presence of ethyl aliphatic groups in the constituent polymers. The interaction led to the formation of both intermolecular H-bonds between the sugar moieties (Figure 5a). These underlying weak chemical interactions may not cause a structural change in the polymers but may initiate aggregation of the aliphatic chains creating dense localized regions not characteristic of the bulk polymers. In addition, ALG-induced intramolecular hydrogen bonding in HEC and vice versa may influence the hydration process of the polymer matrix. This energy stabilization along with the matrix entanglement may provide a prolonged nicotine release along with reduced burst release as compared to alginate alone. 

We further screened ALG–HEC interactions for two divalent cations (Ca^2+^ and Ba^2+^) using SLAS, wherein the ion probes were placed within the van der Waals surface of the polymeric chains as shown in Figure 5a,b. The component binding energies listed in Equations (5), (8), (10) and (11) confirmed that the ALG–HEC–Ba^2+^ formed a more thermodynamically stable conformer than ALG–HEC–Ca^2+^. Interestingly, the polymer chains formed an ordered secondary structure outlined by the presence of cation binding sites as ordered arrays and within the polysaccharide fragments. These energy optimizations were supported by the electrostatic forces while the van der Waals interactions and the torsion angle energies destabilized the cation-crosslinked molecules. The geometrical conformation of the polymer chains and the steric organization of the cations conformed the role of stereospecificity, ligand spacing and position of the coordination shell for the cation interaction display (Figure 5). A close look at Figure 5 revealed that Ba^2+^ provided significantly close packed 3D architecture as compared to Ca^2+^ and hence increased the tendency of the polymer network to form intraglycosidic hydrogen bonds and an increase in hydration energy. In essence, the torsional strains, cavity formation contribution and hydration factors together contributed to complexation energies. This behavior of the Ca^2+^ and Ba^2+^ was also in agreement with experimental data in the case of ALG–HEC gelispheres where the corrugated polysaccharide chains might “offer several oxygen atoms whose stereochemical arrangement fits into the coordination sphere” of the cations resulting in prolonged release of nicotine from gelisphere matrices.

#### 3.7.3. 3D Computational Modeling for Polymer–Polymer Complexes

The measured differences in the flowability and friability properties and texture profile of external matrices were elucidated by randomly disposing PLGA or PEO or PLGA/PEO around HPMC to form HPMC–PLGA, HPMC–PEO and PLGA/HPMC/PEO.

*E*_HPMC_ = 49.713*V*_∑_ = 2.089*V*_b_ + 18.821*V*_θ_ + 22.360*V*_φ_ + 6.776*V*_ij_ − 0.335*V*_hb_ (12)

*E*_PLGA_ = 2.021*V*_∑_ = 0.522*V*_b_ + 4.905*V*_θ_ + 2.575*V*_φ_ − 5.982*V*_ij_ (13)

*E*_PEO_ = 29.483*V*_∑_ = 0.643*V*_b_ + 6.092*V*_θ_ + 15.675*V*_φ_ + 7.072*V*_ij_ (14)

*E*_PLGA–HPMC_ = 32.098*V*_∑_ = 2.366*V*_b_ + 21.460*V*_θ_ + 26.393*V*_φ_ − 17.375*V*_ij_ − 0.747*V*_hb_ (15)

[Δ*E*_BINDING_ = −19.636 kcal/mol]

*E*_PLGA–PEO_ = 11.572*V*_∑_ = 1.240*V*_b_ + 8.231*V*_θ_ + 18.895*V*_φ_ − 16.794*V*_ij_ (16)

[Δ*E*_BINDING_ = −19.932 kcal/mol]

*E*_HPMC–PEO_ = 53.607*V*_∑_ = 2.540*V*_b_ + 17.290*V*_θ_ + 42.725*V*_φ_ − 7.945*V*_ij_ − 1.003*V*_hb_ (17)

[Δ*E*_BINDING_ = −25.236 kcal/mol]

*E*_PLGA–HPMC–PEO_ = 40.662*V*_∑_ = 3.043*V*_b_ + 25.015*V*_θ_ + 40.473*V*_φ_ − 26.637*V*_ij_ − 1.233*V*_hb_ (18)

[Δ*E*_BINDING_ = −40.555 kcal/mol]

It is evident from Equations (12)–(18) that the formation of HPMC–PLGA, PLGA–PEO, HPMC–PEO and PLGA/HPMC/PEO (in vacuum) were accompanied by energy stabilization of −19.636 kcal/mol, −19.932 kcal/mol, −25.236 kcal/mol and −40.555 kcal/mol, respectively. Since all four molecular complexes displayed –ve energy of binding, it can be estimated that the polymers demonstrated compatibility and stability to form a polymeric blend in a dried state [22]. 

**Figure 6:**
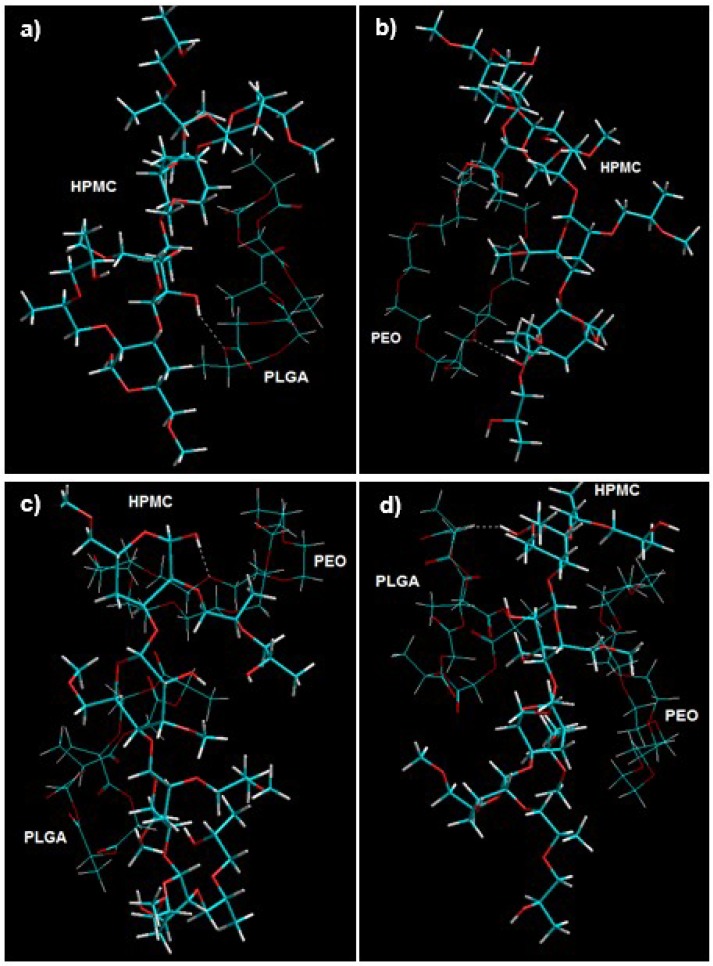
Visualization of geometrical preference of (**a**) PLGA in complexation with HPMC; (**b**) PEO in complexation with HPMC; (**c**) PLGA–HPMC–PEO tripolymeric comples with PEO H-bonded to HPMC; and (**d**) PLGA–HPMC–PEO tripolymeric complex with PLGA H-bonded to HPMC after molecular mechanics simulations. Color codes: C (cyan), O (red), N (blue), and H (white).

The chemical interactions within the polymeric blends were primarily represented by non-bonding interactions (van der Waals forces) and were accountable for the for mechanical strength and flow characteristics of the composites. This energy minimization involving HPMC demonstrated extensive rotations among the monosaccharide residues leading to the formation of a strained architecture which was eventually relieved by angle adjustments and bond length, resulting in H-bonding within the PLGA–HPMC and PEO–HPMC composites (Figure 6). As reported earlier in this paper, PLGA–HPMC–PEO produced highly dense compressed discs due to high angle of repose and flowability. This could be due to the interactions of aliphatic groups of PLGA/PEO with pendent groups of HPMC → formation of unfavorable regions → large steric modulations and overcoming of torsional barriers → large accessible potential energy surface → formation of a polymeric matrix. This became even more applicable when the matrices were hydrated. Given the high torsional strain within the HPMC matrices, it may induce degradation of the polymeric matrix in order to acquire energy stabilization upon hydration. The movement of water molecules inside the torsional strains may in turn lead to an early release of drug molecules. This corroborates with the experimental results wherein rapid release rates were observed from PEO and HPMC matrices (Figure 3). This torsional restraint relaxation during hydration also led to the decrease in BHN values in HPMC and PEO matrices as compared to PLGA matrices (Figure 1).

## 4. Conclusions

The intended drug delivery system that this study sought to develop was one that could provide sustained zero-order release of nicotine from a crosslinked polyspheric system. This would allow for the attainment of constant drug levels at the site of action and minimize adverse effects associated with fluctuating drug levels, as well as optimizing patient therapy and compliance. PLGA matrices incorporating calcium barium-crosslinked alginate-hydroxyethylcellulose gelispheres offered minimal rates of matrix degradation and successfully retarded nicotine release, leading to the achieved zero-order release for 50 days following exposure to simulated CSF. The computational modeling methods that were used for the prediction of preferred molecular conformations of the drug–polymer and polymer-polymer complexes using force field minimizations and the modes of interaction corroborated well the experimental results. The developed drug delivery system displays a great potential for use in the treatment of Parkinson’s disease.

## Figures and Tables

**Table 1:** Polymeric compositions of the various external matrix formulations. Friability results for polymeric matrices expressed as % weight loss after 4 (% WL_4_) and 20 (% WL_20_) minutes in a friabilator at 25 rpm. HPMC: hydroxypropylmethylcellulose; PEO: polyethylene oxide; PLGA: poly(lactic-*co*-glycolic) acid.

Formulation Code	HPMC (mg)	PEO (mg)	PLGA (mg)	% WL_4_	% WL_20_
HPMC	500	-	-	0.081	0.307
PEO	-	500	-	0.101	0.133
PLGA	-	-	500	0.000	0.026
HPMC-PEO	250	250	-	0.050	0.107
HPMC-PLGA	250	-	250	0.051	0.064
PEO-PLGA	-	250	250	0.038	0.102
HPMC-PEO-PLGA	166	166	166	0.025	0.051

**Table 2:** Calculated molecular attributes of the complexes involving alginate (ALG) and nicotine (NCT).

Structure	Molecular Attributes		
	Surface Area (grid)	Volume	Surface-To-Volume Ratio
ALG	828.99	1518.13	0.546
NCT^2 #^	603.16	1088.42	0.554
ALG–NCT^2 #^	1026.16	2273.41	0.451

ΔRef = Ref_(HostGuest)_ – Ref_(Host)_ – Ref_(Guest)_. ^#^ Data represent two molecules of nicotine.

## Supplementary Materials

The following are available online at http://www.mdpi.com/1999-4923/10/4/233/s1, Figure S1: Image processing of micrograph of a PLGA scaffold: (**a**) BLURimage: blurred SEMimage; (**b**) CQimage: colorquantized image of BLURimage; (**c**) UniHisto: uniform histogram of CQimage; (**d**) UniHistoSep: separated uniform histogram of CQimage after image processing; (**e**) NonUniHisto: non-uniform histogram of SEMimage before image processing; and (**f**) NonUniHistoSep: separated non-uniform histogram of SEMimage before image processing. Note that each color channel is only capable of highlighting most of the pores, but not all.
