# Supplementary material for: In Vitro and In Silico Analyses of Nicotine Release from a Gelisphere-Loaded Compressed Polymeric Matrix for Potential Parkinson’s Disease Interventions
Source: Pharmaceutics. 2018 Nov 15;10(4):233. doi: 10.3390/pharmaceutics10040233 (PMC6320845; doi:10.3390/pharmaceutics10040233)
Supplement: Supplementary file 1 [file pharmaceutics-10-00233-s001.pdf]

pharmaceutics

Supplementary Materials: In Vitro and In Silico Analyses of Nicotine Release from a Gelisphere-Loaded Compressed Polymeric Matrix for Potential Parkinson's Disease Interventions

Pradeep Kumar, Yahya E. Choonara, Lisa C. du Toit, Neha Singh and Viness Pillay

Image processing using Mathematica™ 8.0

SEM TIFF images were processed using Mathematica™ 8.0 (Wolfram Research, Champaign, IL) as described in the Results section. Initially, images were cropped to restrict image content to that of the scaffold. The whole analysis was performed on a HP Pavilion dv5-1102TU with an Intel Pentium Dual-Core (2.0 GHz, 3 GB RAM) running Microsoft Vista Basic.

Image processing algorithm for polymeric scaffolds

According to Wolfram Research Inc., "*Mathematica* provides broad and deep built-in support for both programmatic and interactive modern industrial-strength image processing—fully integrated with *Mathematica*'s powerful mathematical and algorithmic capabilities. *Mathematica*'s unique symbolic architecture and notebook paradigm allow images in visual form to be included and manipulated directly both interactively and in programs." [http://reference.wolfram.com/mathematica/guide/ImageProcessing.html]. Here we used Mathematica for pore uniformity and pore distribution. Gray level images show a complex structure in which, either an uneven illumination, or a lack of contrast or other sources of noise, hide the real pattern. In order to carry out the pore analysis, pore edges were extracted from the gray level images and histographical analysis was carried out for the modified images (Figure Xa-Xf).

Sixteen-bit greyscale images obtained by SEM polymeric scaffolds were low in contrast (Fig. 2). This was mostly due to the non-uniform gold coating. In addition, the background was uneven due to uneven illumination across the field of view. Since thresholding of such images were unable to capture all of the required details, we employed some image-processing steps to even out the background and increase contrast between the pores and the rest of the image. Images in TIFF format were imported as shown in expression 1 (Exp 1) and converted to Mathematica™ 8.0 format:

```
Exp1: SEMimage = Import["X:\\directory\\folder\\filename.tif"];
```

The first processing step, blurring the image, gave a blurred version of image making the image unfocused which can be obtained by convolving the image with a low pass filter. In this work, the amount of blurring is increased by increasing the pixel radius (r) to 10 without compromising image detail. This was achieved in Mathematica™ 8.0 by applying a custom blurring function (Exp 2) giving Figure S1a:

```
Exp 2: BLURimage = Blur[SEMimage,10];
```

After blurring the image, the next step was to ColorQuantize the blurred image at 5 which provided an approximation to image that uses only 5 distinct colors. This was achieved in Mathematica™ 8.0 by applying a custom coloequantize function (Exp 3) giving Figure S1b:

```
Exp 3: CQimage = ColorQuantize[BLURimage,5];
```

The third step consisted of analysing the colorquantized image by plotting a histogram. This default function plots a histogram of the pixel levels for each channel in image. Furthermore, separate histograms for each color channel were also constructed. This was achieved in Mathematica™ 8.0 by applying the custom ImageHistogram functions for default and separated algorithms as displayed in

Exp 4 and Exp 5 giving Figure S1c and Figure S1d, respectively:

```
Exp 4: UniHisto = ImageHistogram[CQimage];
Exp 5: UniHistoSep = ImageHistogram[CQimage,Appearance→"Separated"];
```

Additionally, the ImageHistograms were also constructed for the original unprocessed image, using Exp 6 and Exp 7 giving Figure S1e and Figure S1f, for comparative analysis with the processed one.

```
Exp 6: NonUniHisto = ImageHistogram[SEMimage];
Exp7:NonUniHistoSep=ImageHistogram[SEMimage,Appearance→"Separated"]
```

The comparative analysis before (Figure S1e, S1f) and after (Figure S1c, S1d) image processing revealed the striking effect of these steps on image histogram's clarity such that the presence of pore uniformity and distribution is more obvious. The PLGA scaffold can thus be seen as for having a uniformly distributed and uniformly sized porous structure. Different volume in terms of size and depth of the pores generated different color patterns in the histograms which are in line with the porosity-erosion experimental results.

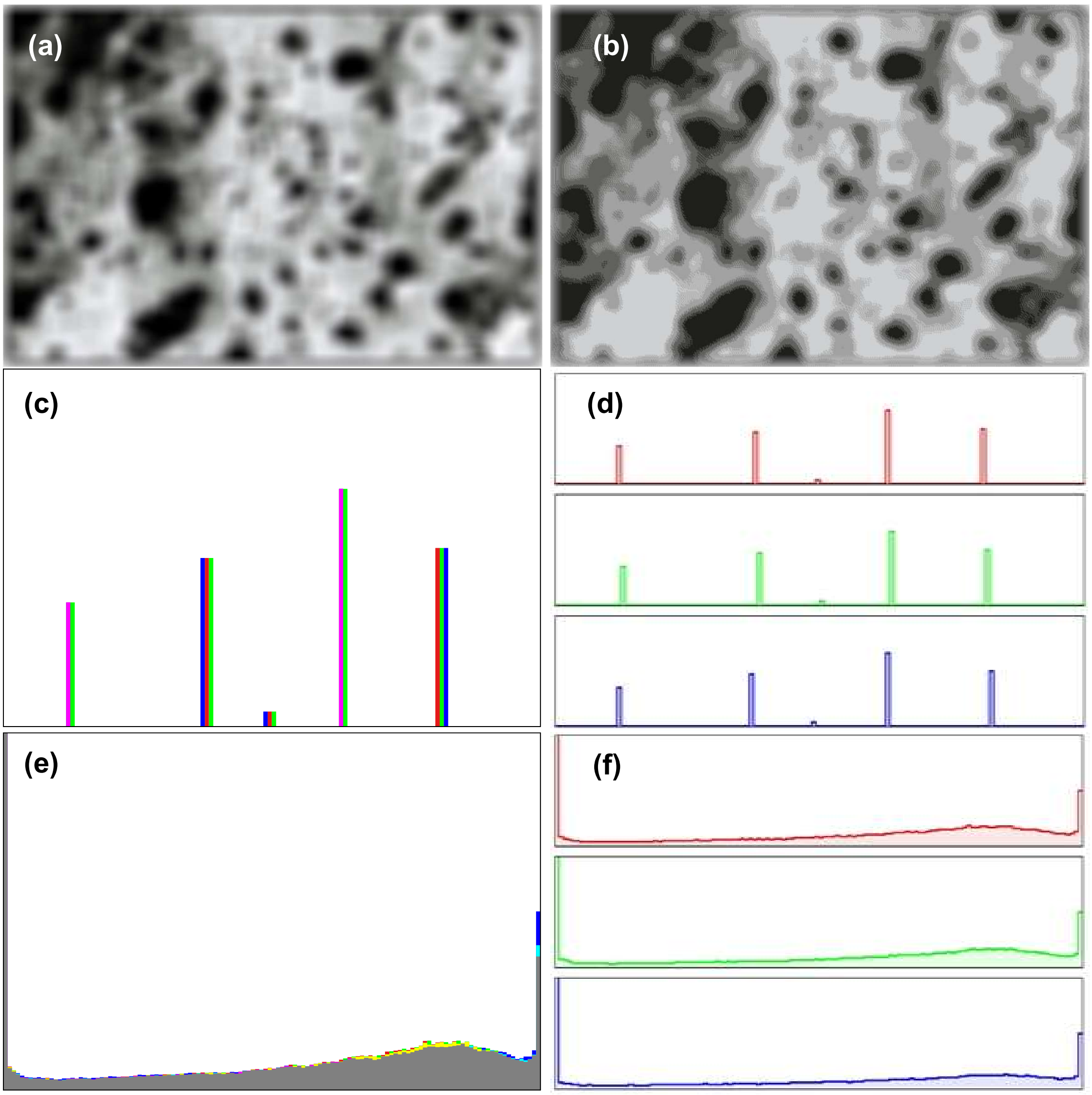

Figure S1: Image processing of micrograph of a PLGA scaffold: (**a**) BLURimage: blurred SEMimage; (**b**) CQimage: colorquantized image of BLURimage; (**c**) UniHisto: uniform histogram of CQimage and (**d**) UniHistoSep: separated uniform histogram of CQimage after image processing; (**e**) NonUniHisto: non-uniform histogram of SEMimage before image processing; (**f**) NonUniHistoSep: separated non-uniform histogram of SEMimage before image processing. Note that each color channel is only capable of highlighting most of the pores, but not all.
